# Paraneoplastic morphea with prominent mucin deposition

**DOI:** 10.1016/j.jdcr.2023.09.015

**Published:** 2023-09-26

**Authors:** Ahmad B. Shahin, Adina Greene, Craig B. Reeder, Collin M. Costello, Mark R. Pittelkow, David J. DiCaudo

**Affiliations:** a Mayo Clinic Alix School of Medicine, Scottsdale, Arizona; b University of Arizona College of Medicine, Phoenix, Arizona; c Department of Dermatology, Mayo Clinic, Scottsdale, Arizona; d Division of Hematology/Oncology, Mayo Clinic, Phoenix, Arizona; e Department of Laboratory Medicine and Pathology, Mayo Clinic, Scottsdale, Arizona

**Keywords:** cutaneous mucinosis, morphea, myeloma, paraneoplastic, scleroderma

## Introduction

Morphea characteristically shows sclerosis of dermal collagen on biopsy.^1^^,^^2^ Conspicuous mucin deposition is only rarely seen in morphea lesions. We report a patient with smoldering myeloma who developed paraneoplastic morphea with prominent mucin deposition within the morphea lesions.

## Case report

**Fig 1:**
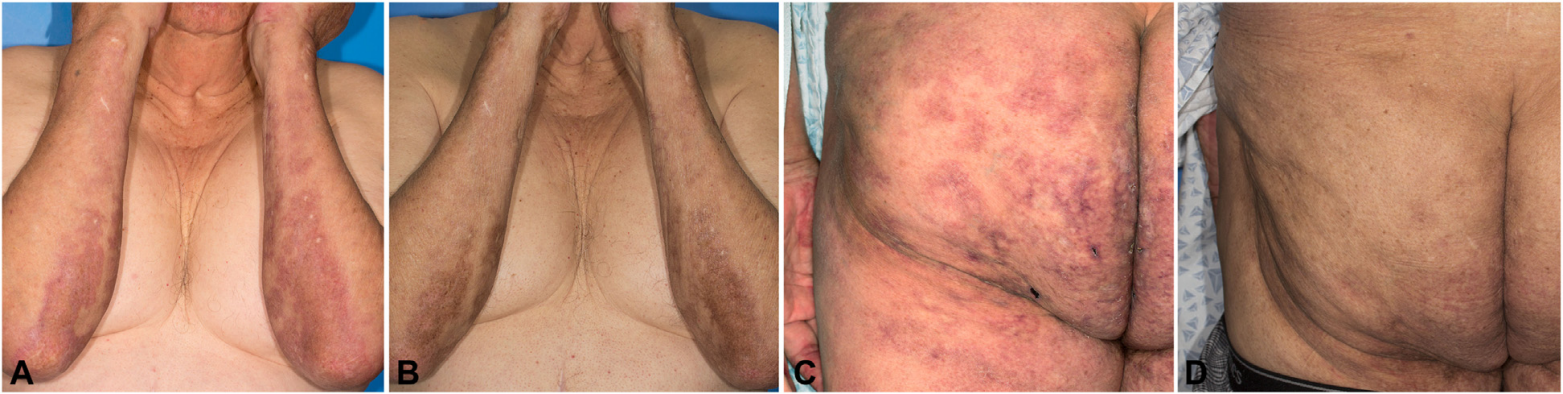
Initial skin findings and response to lenalidomide (**A** and **B**), bilateral forearms and (**C** and **D**), *left* buttock (**A** and **C**), pretreatment (**B** and **D**), 15 months after treatment.

An 80-year-old male with history of immunoglobulin G kappa smoldering myeloma diagnosed 13 years ago presented to dermatology. He had a 2-year history of dusky, erythematous, indurated patches on the lower abdomen and back, as well as a more hyperpigmented plaque on the right upper calf. The patient had a history of similar lesions 6 years prior that were initially diagnosed as tumid lupus but ultimately were identified as paraneoplastic mucinosis. The cutaneous mucinosis improved partially from topical corticosteroid therapy, but experienced complete resolution after 9 months of lenalidomide therapy (Fig 1). He underwent 4 cycles of lenalidomide, 15 mg daily for 21 days, followed by 7 days off. However, after experiencing fatigue on that regimen, he was transitioned to a dose of 10 mg for his last 3 cycles.

**Fig 2:**
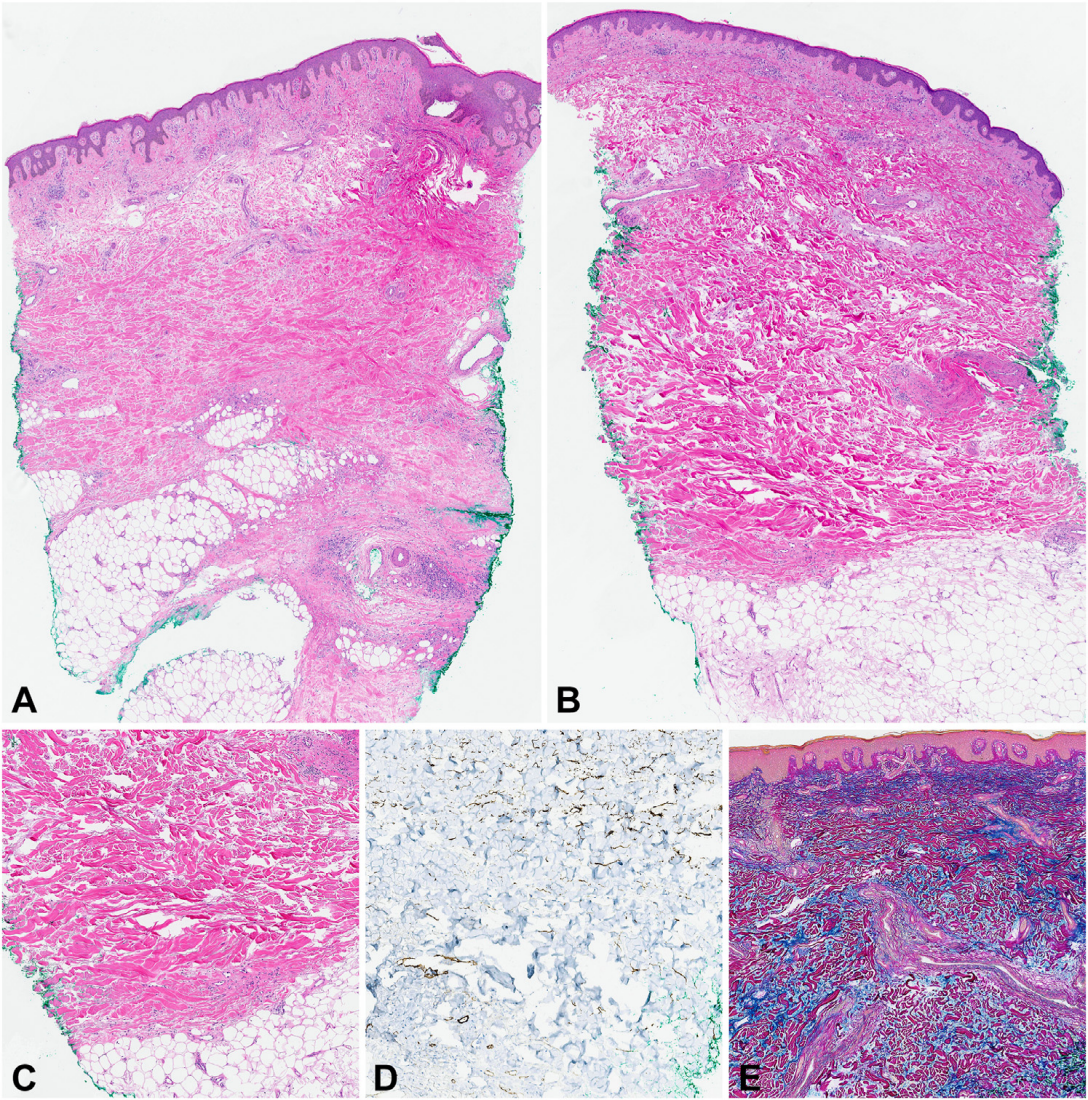
**A,***Right* calf skin biopsy showing dermal and subcutaneous sclerosis (hematoxylin-eosin stain, 1.6× magnification, digital scan at 40×). **B,***Right* side of the back skin biopsy with splaying and thickening of the dermal collagen bundles (hematoxylin-eosin stain, 1.6× magnification, digital scan at 40×). **C,***Right* side of the back skin biopsy demonstrating dermal sclerosis (hematoxylin-eosin stain, 8× magnification; digital scan at 40×). **D,** CD34 stain illustrating loss of CD34^+^ dermal dendrocytes (13× magnification; digital scan at 40×). **E,** Colloidal iron stain showing increased dermal mucin between the splayed collagen bundles (10× magnification; digital scan at 40×).

Biopsy of the right calf demonstrated dermal sclerosis with hyalinized collagen bundles extending into the subcutis. CD34^+^ dermal dendrocytes were absent in the zones of sclerosis. The biopsy of the right side of the back similarly showed dermal sclerosis but also had abundant interstitial deposits of dermal mucin (Fig 2). CD34^+^ dermal dendrocytes were diminished in the sclerotic zone. The abundant interstitial dermal mucin was confirmed with the colloidal iron stain.

On further workup, persistent immunoglobulin G monoclonal gammopathy, kappa restricted, was detected; laboratory tests were otherwise normal. No other evidence of systemic mucinosis was detected clinically or on whole-body computed tomography. At that time, a morphea/lupus erythematosus overlap syndrome and mixed connective tissue disease were considered in the histopathologic differential diagnosis due to the combined histopathologic features. Features suggestive of lupus included focal periadnexal lymphocytic inflammation (not shown) and the abundant interstitial dermal mucin. However, a paraneoplastic process was favored, given the immunoglobulin G gammopathy and previous response to lenalidomide. The patient's smoldering myeloma was again treated with lenalidomide, and the cutaneous lesions resolved over 8 months.

Six months after the resolution of these lesions, the patient developed painful, violaceous, ulcerated lesions on the dorsal and lateral feet, ankles, and left upper posterior calf-popliteal region, suspicious of livedoid vasculopathy. Dermatopathology and direct immunofluorescence confirmed livedoid vasculopathy. The patient was started on apixaban, and bone marrow biopsy was performed. A new large B-cell lymphoma, as well as recurrent smoldering myeloma, was identified. The patient was initiated on a reduced dose regimen of rituximab plus cyclophosphamide, doxorubicin, vincristine, and prednisone and has not had any recurrence of paraneoplastic morphea.

## Discussion

This distinctive case is exceptional for its paraneoplastic associations and unusual histopathologic finding of dermal mucin. Sclerodermatous processes have previously been reported in association with multiple myeloma and monoclonal gammopathy but without mucinous deposits.^3^ Prominent mucin deposits are a rare finding in morphea. A previous report of linear morphea described mucin deposits localized to the deep reticular dermis and interlobular septa of the subcutis.^4^ Prominent mucin deposition has also been described in a case of solitary morphea profunda^5^ and sclerodermoid graft-versus-host disease.^6^

Potential pathogenic mechanisms underlying mucin deposition include stimulation of fibroblasts by growth factor-β leading to overproduction of glycosaminoglycans, hyaluronic acid, and collagen, characteristic of mucinoses.^4^^,^^6^^,^^7^ In 1994, Rongioletti et al demonstrated mucin deposition in each of 20 cases of systemic scleroderma.^8^ Nevertheless, conspicuous mucin on routine stains is uncommon in morphea and systemic scleroderma.

Paraneoplastic morphea has rarely been reported. Scleroderma-like changes without mucinous deposits have been reported in association with multiple myeloma and monoclonal gammopathy.^3^ An underlying adenocarcinoma of pancreatobiliary origin was found in a patient manifesting paraneoplastic plaque-like cutaneous mucinosis.^9^

Our patient's cutaneous manifestations conformed with a paraneoplastic process fulfilling many of Curth paraneoplastic criteria.^10^ Although the initial smoldering myeloma preceded the patient's first paraneoplastic mucinosis, both responded to lenalidomide. When the patient developed paraneoplastic morphea with mucin, 5 of 6 Curth criteria were met, (1) the smoldering myeloma and skin lesions developed simultaneously, (2) they had a parallel course, (3) there was no associated genetic syndrome, (4) morphea with mucin is rare in the general population, and (5) the dermatosis and the myeloma responded to lenalidomide. Though topical corticosteroids offered partial benefit, treatment of smoldering myeloma with lenalidomide repeatedly provided optimal therapy and clearance of the cutaneous paraneoplastic lesions. Recognizing the rare presentation of mucinosis as a paraneoplastic process facilitates appropriate and timely treatment of the cutaneous lesions, as demonstrated in our case.

Concurrent or sequential cutaneous paraneoplastic disorders have very rarely been reported. During our patient's third recurrence of smoldering myeloma, he developed large B-cell lymphoma and paraneoplastic livedoid vasculopathy.

Rare cases of lymphoma have also been reported in patients who present with livedoid vasculopathy. Consideration of a new malignancy should be entertained and pursued with the onset of additional distinct paraneoplastic cutaneous disease.^11^

## Conclusion

Mucin deposition rarely presents in lesions of morphea. In our patient, the morphea lesions occurred as a paraneoplastic phenomenon and resolved with treatment of the underlying malignancy. Though rare, the development of sequential cutaneous paraneoplastic conditions warrants recognition and should drive further evaluation for recurrence or new primary malignancy.

## Conflicts of interest

None disclosed.
